# Characterization of mechanical behavior of a porcine pulmonary artery strip using a randomized uniaxial stretch and stretch-rate protocol

**DOI:** 10.1186/1475-925X-7-4

**Published:** 2008-01-23

**Authors:** Choon-Sik Jhun, John C Criscione

**Affiliations:** 1 Department of Biomedical Engineering, Texas A&M University, College Station, TX, 77843-3120, USA

## Abstract

**Background:**

Much of the experimental work in soft tissue mechanics has been focused on fitting approximate relations for specific tissue types from aggregate data on multiple samples of the tissue. Such relations are needed for modeling applications and have reasonable predictability – especially given the natural variance in specimens. There is, however, much theoretical and experimental work to be done in determining constitutive behaviors for particular specimens and tissues. In so doing, it may be possible to exploit the natural variation in tissue ultrastructure – so to relate ultrastructure composition to tissue behavior. Thus, this study focuses on an experimental method for determining constitutive behaviors and illustrates the method with analysis of a porcine pulmonary artery strip. The method characterizes the elastic part of the response (implicitly in terms of stretch) and the inelastic part in terms of short term stretch history (i.e., stretch-rate) *H*_*t*2_, longer term stretch history *H*_*t*1_, and time since the start of testing *T*.

**Methods:**

A uniaxial testing protocol with a random stretch and random stretch-rate was developed. The average stress at a particular stretch was chosen as the hyperelastic stress response, and deviation from the mean at this particular stretch is chosen as the inelastic deviation. Multivariable Linear Regression Analysis (MLRA) was utilized to verify if *H*_*t*2_, *H*_*t*1_, and *T *are important factors for characterizing the inelastic deviation. For acquiring *H*_*t*2 _and *H*_*t*1_, an integral function type of stretch history was employed with time constants chosen from the relaxation spectrum of an identical size strip from the same tissue with the same orientation. Finally, statistical models that characterize the inelasticity were developed at various, nominal values of stretch, and their predictive capability was examined.

**Results:**

Inelastic deviation from hyperelasticity was high (31%) for low stretch and declined significantly with increasing stretch to a nadir of 3.6% for a stretch of 1.7. The inelastic deviation then increased with increasing stretch at the same point in the stress-strain curve where stiffness began to increase strikingly. MLRA showed that *T *is a major inelastic parameter at low deformation. For moderate and high deformations, *H*_*t*2 _and *H*_*t*1 _were dominant.

**Discussion:**

A randomized uniaxial testing protocol was applied to a strip of porcine pulmonary artery to characterize the elasticity and inelasticity of a soft tissue. We were successful in determining the elastic response and the factors that gave rise to the inelastic deviation. This investigation seeks methods to better define, phenomenologically, the elastic and inelastic behavior of soft tissues.

## Introduction

A great challenge in biomechanics, and its related disciplines such as mechanobiology, is the determination of mechanical constitutive behaviors for tissues that undergo high strain. Even for homogeneous, single phase, rubber-like materials with behavior that is nearly isotropic and hyperelastic, the determination of constitutive behaviors has been ambiguous and problematic [1]. For multi-phase, heterogeneous tissues with behavior that is anisotropic and visco-elastic, much theoretical and experimental work remains [2], and this work is a small step toward improving experimental methods to delineate tissue behavior.

There are, of course, approximate constitutive relations for most tissue types that have been validated with fits to experimental data and that have enabled modeling applications in biomechanics. Rather than integrate tests from multiple specimens to yield a generalized relation, this work is focused on a methodology to study individual specimen variations. Our reason for doing so is to exploit the natural variation in tissue structure to better understand the structure-function relationship. Each specimen has differing amounts of elastin, collagen, water, etc.; and with experimental methods that delineate rather than aggregate behavior, we seek to relate ultrastructure parameters to tissue response parameters. The ultimate goal of this work, albeit decades away, is to determine how ultrastructure maps to tissue behavior.

Toward this end, we developed a random stretch and random stretch-rate protocol for a one-dimensional sample (longitudinal strip from a porcine pulmonary artery). The average stress at a particular stretch is defined as the elastic or hyperelastic part of the response and the deviation from this average is defined as the inelastic part of the response. The inelastic response is assumed to be a function of three variables: stretch rate related, short tem stretch history *H*_*t*2_; longer term stretch history *H*_*t*1_; and time *T *since the start of the test protocol. At a particular stretch value, the inelastic response is assumed to be a linear function of these variables and dependence is found by Multivariable Linear Regression Analysis (MLRA). A randomized testing protocol eliminates any potential correlation among *H*_*t*2_, *H*_*t*1_, and *T *that conventional cyclic testing cannot. This randomness enables the use of MLRA.

Hoffman and Grigg have introduced the advantage of application of random stress stimuli to the soft tissue [3]. The difference here is that stretch herein, rather than stress therein, is the independent variable that is randomized. Moreover, we seek to define and determine the elastic and inelastic parts of the response at particular chosen values of strain rather than fit behavior for the range tested. Though, there are many time-dependent theories that can capture or fit inelastic behaviors, such as, linear/nonlinear viscoelastic [4-10], poroviscoelastic [11,12], and biphasic [13-16]., this study is focused on experimental methodology for hyperelastic constitutive behaviors.

### Remark on Hyperelasticity

We often mix the terms elastic and hyperelastic when referring to material behavior and certain aspects of the stress response. Conceptually, one can think about elasticity (i.e., stress as a function of stretch or strain) as different than hyperelasticity (i.e., strain energy as a function of stretch or strain); yet in fact, elastic behavior must be hyperelastic – or else it violates the first law of thermodynamics. This is easily shown by considering a closed, cyclic process involving an *elastic *material. An elastic material does not have viscous losses, and the work of the reverse path (or cycle) is the opposite of the forward path (or cycle). This is a direct result of the stress-power law. If the work done is zero for all closed paths, then there exists a state function (strain energy) that represents the storage, in a conservative manner, of work done on the material. This is hyperelasticity wherewith the constitutive law can be defined by its strain energy function and the stress is given by the derivative of the strain energy with respect to the strain. If the work done is not zero for all closed paths (i.e., non-hyperelastic), then choose one such path and go around the cycle in the direction that produces work, and then build a perpetual motion machine of the first kind. It is quite easy (in two and three dimensions) to fit an elastic model that violates the first law [17]; and yet it is impossible for a hyperelastic model to violate the first law. To be explicit: 1) if an elastic model satisfies the first law then it can be expressed in a hyperelastic form, and 2) if an elastic model cannot be expressed in a hyperelastic form, then it violates the first law. When we refer to elastic behavior as hyperelastic, we are only excluding behavior that violates the first law of thermodynamics.

## Methods

**Figure 1:**
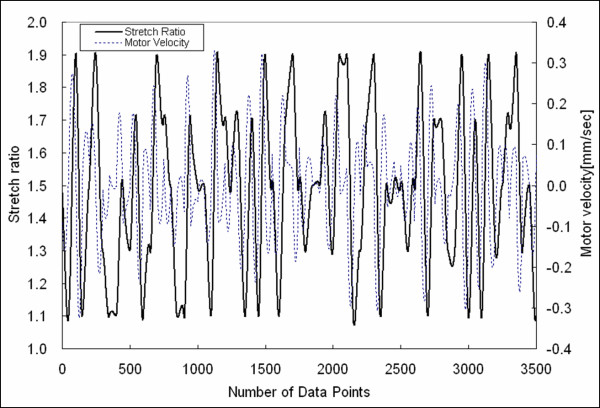
Stretch-controlled protocol showing the stretch and velocity of one of the actuators versus time.

A uniaxial testing protocol wherein the stretch and stretch rate were randomized was developed to better delineate the average stress vs. stretch response of soft tissue. The randomized testing protocol was achieved by defining 71 time points or nodes and then randomly choosing (at the nodes) nominal stretch values of *λ *= 1.1, 1.3, 1.5, 1.7, or 1.9 and stretch-rate values in the range of [-5.26 s^-1^, 5.33 s^-1^]. With 71 time nodes there are 70 intervals, and in each interval we defined 50 points. The stretch value at points between nodes was obtained from the nodal stretches and stretch-rates by use of cubic Hermite interpolation in time, so to achieve C^1 ^continuity across intervals [18]. Figure 1 displays the stretch versus time of the stretching protocol. Upon inputting the reference length of the sample, the stretch versus time protocol was converted to displacement versus time and fed to opposing linear actuators (both ends of the sample were moved in opposite directions, so to double the stretch-rate range).

This stretching protocol was accomplished with custom LabVIEW (National Instruments, Inc) codes. The stretch values were chosen to cover the linear and non-linear portions of the stress-stretch response of the tissue, without damaging the tissue. The stretch-rate range was determined by the upper and lower bound for the speed of the actuators (CMA 25CCCL DC servo motor, max speed = 0.4 mm/s, Newport, Inc). The actuators were controlled by the Universal Motion Controller/Driver (ESP7000, Newport, Inc). The total testing time was 5600 seconds.

**Figure 2:**
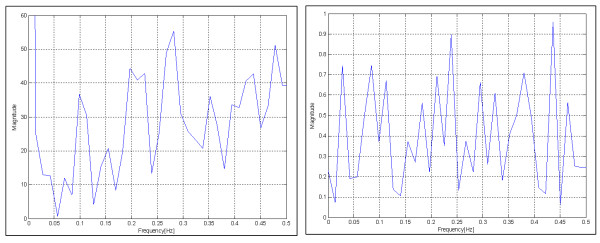
Power spectra for stretch (left) and stretch rate (right). Although not uniform and flat, there is broad spectral distribution with noise dominating any systematic trends.

Fourier spectral analysis was performed on both randomized stretch and randomized stretch rate profiles to validate their randomness (Figure 2). The power spectrum is not ideally uniform across the spectrum, but it has power distributed across the spectrum with noise dominating any systematic trends.

The pulmonary artery from an adult swine was obtained from a local slaughter house immediately after slaughter. Two identical strips (25.4 mm × 2.5 mm × 2 mm) were cut from the porcine pulmonary artery (one was used for the stretch protocol and one for the relaxation analysis), preserved in chilled phosphate buffered saline (PBS) and tested at room temperature (approximately 23°C). We mounted a specimen by using custom-built alligator type grips and no slip was detected during the test. The stretch ratios were calculated based on a gauge length which is a grip-to-grip displacement. (In general, grip-to-grip strain is greater than average strain measured by optical strain measurement because of edge effects and local deformation at the tip of the grip. Since the length of our specimen is relatively long relative to its width, 10:1; and since this work is focused on archiving and discussing the methodology as opposed to archiving the behavior of a particular, now decomposed, sample, we neglected edge effects.) The average force at a particular stretch was defined as the hyperelastic response because it would be the best guess for the response if the stretch rate and stretch history were unknown or neglected – as per the assumption of elasticity. Inelastic deviation was defined and measured as the deviation from the average force at each nominal stretch ratio.

To characterize the inelastic deviation *D *at a particular stretch value, we assumed that it is a function of the following form.

$$D=D(T,H_{t_{2}},H_{t_{1}})$$

To quantify inelastic behavior, we assumed the functional form shown in equation (2) where *F*_*M *_and *F*_*W *_are measured and average force, respectively at a particular stretch.

*F*_*M *_= *F*_*W *_± *D*(*H*_*t*2_, *H*_*t*1_, *T*)

To explicitly represent the limits of experimental methodologies, we included an unknown factor *D*_*e *_that represents additional inelastic deviation (or random error) beyond that which is represented by *D*_*Ht*2_, *D*_*Ht*1_, and *D*_*T *_or deviation due to *H*_*t*2_, *H*_*t*1_, and *T*, respectively. Hence,

*D *= *D*_*Ht*2 _+ *D*_*Ht*1 _+ *D*_*T *_+ *D*_*e*_

To determine relative uncertainty, we normalized *D *(i.e., standard deviation of measured forces at each stretch ratio) with respect to the average force at a particular stretch and defined the fractional uncertainty *FU*.

$$FU=\frac{D}{F_{W}}$$

### Stretch history

Stretch history is, generally, the deformation versus time for the entire life of the sample up to the current configuration. Such a concept is too all-inclusive to be practical, and yet the mechanical properties of tissues are highly sensitive to prior deformation – i.e., resting, preconditioning, or over-stretching of a sample will significantly perturb the stress-strain response. There are many ways to quantify a stretch history function. The function that we used for stretch history is shown in equation (5). It represents the average stretch for preceding time interval *t*_h_, where *λ*(*t*) is stretch as a function of time, *t*_*N *_is a current time, and *t*_*h *_is a preceding time interval.

$$\begin{array}{cc} H(t)=\frac{\int_{t_{N}-t_{h}}^{t_{N}}\lambda (t)dt}{t_{h}}, & t_{h}>0 \end{array}$$

**Figure 3:**
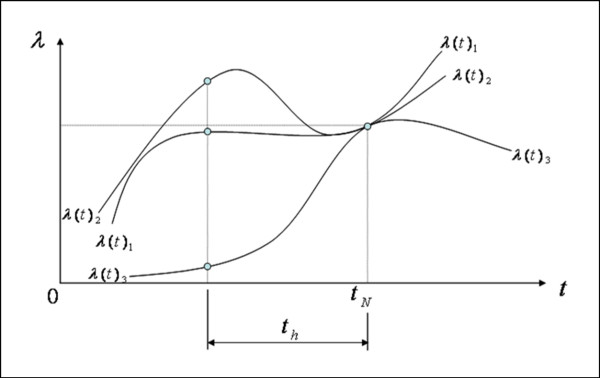
Illustration of various stretch histories with the same stretch and stretch rate at time *t*_*N*_. Although the stretch ratio functions *λ*(*t*)_1_, *λ*(*t*)_2 _and *λ*(*t*)_3 _give the same stretch and stretch rate at time *t*_*N*_, the average stretch ratios for the time duration *t*_*h *_for each are distinctly different.

Note that as *t*_*h *_gets smaller, *H*(*t*) approaches *λ(t*_*N*_) and gives the near term history or instantaneous stretch ratio if *t*_h _≅ 0. On the contrary, if *t*_*h *_gets larger, *H*(*t*) approaches the average stretch ratio (which is *λ *= 1.5 in this study because we set *λ *= 1.0 and *λ *= 2.0 as bounds on a random distribution). Figure 3 displays our stretch history concept. Although stretch functions *λ*(*t*)_1_, *λ*(*t*)_2_, and *λ*(*t*)_3 _give the same stretch and stretch rate at *t*_*N*_, the histories are different.

**Figure 4:**
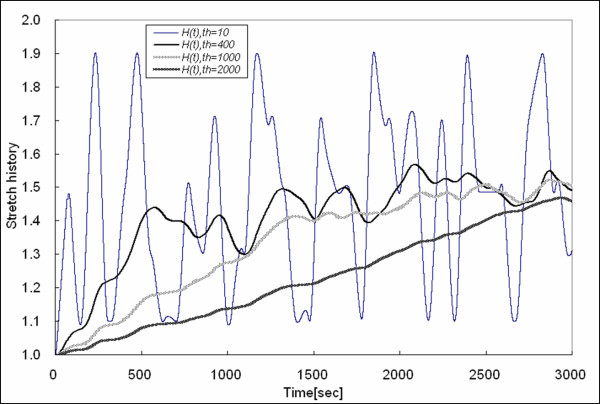
Stretch history obtained by *t*_*h *_= 10, *t*_*h *_= 400, *t*_*h *_= 1000, and *t*_*h *_= 2000 (for 0–3000 sec). When *t*_*h *_is small, the stretch history is nearly the same as the instantaneous stretch, and as *t*_*h *_increases, stretch history reaches a plateau of *λ *= 1.5, the average stretch ratio of a random distribution in the range [1,2].

**Figure 5:**
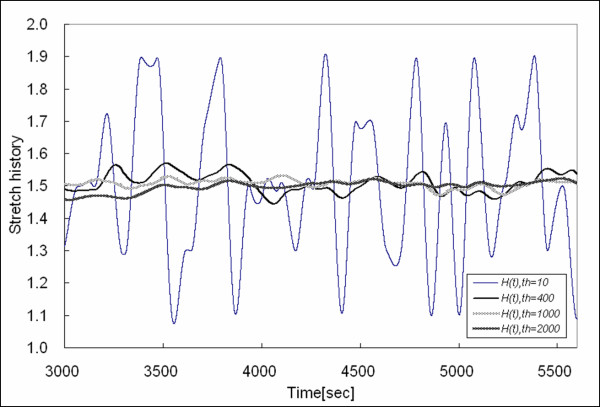
Continuation of Figure 4.

**Figure 6:**
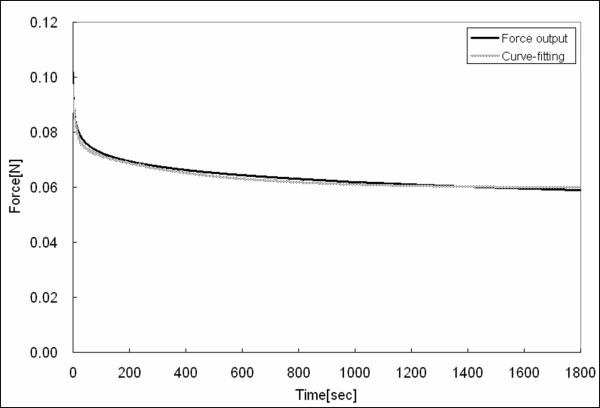
Stress relaxation curve for the tissue and the multi-exponential fit.

Figures 4 and 5 show the stretch history obtained by using *t*_*h *_= 10, 400, 1000, and 2000. Note that the stretch history obtained by *t*_*h *_= 1000 and *t*_*h *_= 2000 approaches the average of *λ *= 1.5. Thus, finding a reasonable *t*_*h *_is important for stretch history to characterize the inelasticity due to history. Upon analyzing the relaxation spectrum for a sample held at a particular stretch (*λ *= 2, max stretch in a random distribution), it is evident that there is short term (fast) relaxation and longer term (slow) relaxation. We fit exponential decays to the stress relaxation curve, and it was fit well by the summation of four exponential functions. The stress relaxation curve is shown in Figure 6, and *F*(*t*) is the force to maintain a step increase in stretch and fit by the multi-exponential decay as follows:

*F*(*t*) = 6.1 + 1.5*e*^-*t*/400 ^+ 0.5*e*^-*t*/30 ^+ 1.3*e*^-*t*/10 ^+ 0.5*e*^-*t*^

**Figure 7:**
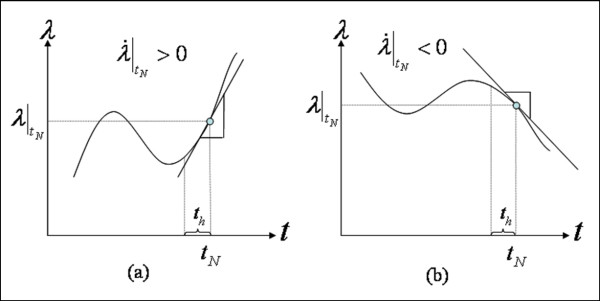
**Relationship between short-term stretch history and stretch rate**. (a) When the stretch rate is positive, the stretch history for *t*_*h *_= 10 sec is less than the current stretch ratio. (b) When the stretch rate is negative, the stretch history for *t*_*h *_= 10 sec is greater than the current stretch ratio.

We consider the two fastest as fast relaxation spectrums (*t*_*h *_= 1, *t*_*h *_= 10) and the two slowest as slow relaxation spectrums (*t*_*h *_= 30, *t*_*h *_= 400), and note that the weights of the exponential functions corresponding to *t*_*h *_= 10 and *t*_*h *_= 400 are greater than the others. Hence, we judiciously chose *t*_*h *_= 10 and *t*_*h *_= 400 for short-term history (*H*_*t*2_) and long-term history (*H*_*t*1_), respectively. Our system could not capture fast deformation histories within 1 second because our lead screw actuators are too slow to deliver a step function in stretch, ideally needed to analyze stress relaxation. Note that the stretch history obtained by *t*_*h *_= 10 is of relatively short duration compared to our acceleration, and it depends, thus, on the direction of approach or stretch rate (see Figure 7) and is roughly the current stretch minus half of the stretch rate times 10 sec. Equation (7) shows the relationship between short term stretch history *H*_*t*2 _and stretch rate $\dot{\lambda }(t)$ when the history is brief relative to the change in history (i.e., stretch acceleration).

$$\begin{array}{llll} \text{if} & {\dot{\lambda }(t)|}_{t_{N}}>0, & H(t)={\frac{\int_{t_{N}-t_{h}}^{t_{N}}\lambda (t)dt}{t_{h}}|}_{t_{h}=10} & <{\lambda (t)|}_{t_{N}}\\ \text{if} & {\dot{\lambda }(t)|}_{t_{N}}<0, & H(t)={\frac{\int_{t_{N}-t_{h}}^{t_{N}}\lambda (t)dt}{t_{h}}|}_{t_{h}=10} & >{\lambda (t)|}_{t_{N}} \end{array}$$

### Multiple linear regression analysis

To characterize inelastic behavior in terms of *H*_*t*2_, *H*_*t*1_, and *T*, multivariable linear regression analysis, goodness-of-fit test and *t*-test were used [19]. Basically, we did a separate statistical analysis for each nominal stretch value. Because each nominal stretch value was retested many times (71 nodes and only 5 possible stretch values), there is a set of force measurements at the same stretch – but each with a different history. *T*, *H*_*t*2_, and *H*_*t*1 _are calculated from the stretching protocol, and *F*_*M *_is measured by the force transducer and captured in synchrony with the stretching protocol. *T*, *F*_*M*_, *H*_*t*2_, and *H*_*t*1 _were grouped together for each stretch ratio. The grouped data for *λ *= 1.1, for example, can be expressed in matrix form as in equation (8) where *n *is the number of data corresponding to stretch ratio *λ *= 1.1.

$$\begin{array}{cccc} {\left[\begin{array}{cccc} D_{1} & T_{1} & {\left(H_{t2}\right)}_{1} & {\left(H_{t1}\right)}_{1}\\ . & . & . & .\\ . & . & . & .\\ . & . & . & .\\ D_{n} & T_{n} & {\left(H_{t2}\right)}_{n} & {\left(H_{t1}\right)}_{n} \end{array}\right]}_{\lambda =1.1} & \text{where} & D_{i}={\left(F_{M}\right)}_{i}-\frac{\sum_{k=1}^{n}{\left(F_{M}\right)}_{k}}{n}, & i=1,2,...,n \end{array}$$

For use in MLRA, the model for inelastic deviation from the mean or elastic part is:

$$\begin{array}{cc} D_{i}=\hat{\alpha }+{\hat{\beta }}_{1}T_{i}+{\hat{\beta }}_{2}{\left(H_{t2}\right)}_{i}+{\hat{\beta }}_{3}{\left(H_{t1}\right)}_{i}+e_{i}, & i=1,2,...,n \end{array}$$

The partial regression coefficients ${\hat{\beta }}_{j}$, *j *= 1, 2 and 3 describe how the independent variables *T*_*i*_, *(H*_*t*2_)_*i*_, and *(H*_*t*1_)_*i *_affect the dependent variable *D*_*i*_. We minimized *e*_*i*_, the errors in fitting, by using a least squares method (LSM) and so obtained $\hat{\alpha }$ and ${\hat{\beta }}_{j}$. To avoid scale-dependency in the partial regression coefficients, we normalized each data set by their magnitudes. Upon fitting the ${\hat{\beta }}_{j}$, we checked ${\bar{R}}^{2}$, the adjusted coefficient of determination to observe how well the derived sample regression line describes the observed variable *D*_*i *_and then examined the significance of each ${\hat{\beta }}_{j}$ using *t*-test. Successive iterations of *t-test *were performed whenever there was/were insignificant variable(s) for a particular stretch. Since we tested a single specimen, we set the level of significance *α *as 0.1 which represents the 90% probability that the partial regression coefficient ${\hat{\beta }}_{j}$ is likely to be contained within the interval. In general, a level of significance *α *is preferably set as 5%.

### Predictive capability

The predictive capability was examined by comparing the predicted inelastic deviations calculated by the derived regression model to the measured inelastic deviations from unused stretch ratios. Only the 71 nodal time points were used to regress the coefficients; yet there were many more times, within the interval between nodes, that the stretch protocol prescribed one of our nominal stretch values of interest (i.e., *λ *= 1.1, 1.3, 1.5, 1.7, or 1.9). Equation (10) was used for the interval prediction where ${\hat{D}}_{fc}$ is critical forecasting interval for the ${\hat{D}}_{f}$ with a 100(1-*α*) confidence, ${\hat{D}}_{f}$ is the predicted value of a certain point, *t*_*c*(*α*/2;*n*-*k*-1) _is a critical *t *value with a degree of freedom *n-k-1 *where *n *and *k *are the number of samples and the number of independent variables, respectively, *s*_*f *_is the standard deviation which consists of variance of *e*_*i *_and variance of ${\hat{D}}_{i}$.

$${\hat{D}}_{fc}={\hat{D}}_{f}\pm t_{c(\alpha /2;n-k-1)}\cdot s_{f}$$

Lastly, to assess the goodness of fit and search for further systematic behavior, we calculated the *deviation of the predication*, *D*_*i *_- ${\hat{D}}_{f}$, which is the difference between observed deviation of a data point *D*_*i *_and point forecasting value ${\hat{D}}_{f}$ obtained by the statistical model for the particular, nominal stretch value. If the deviation of the predication has systematic variation, then it can reasonably be said that there must be other factors (or perhaps higher orders of *H*_*t*2_, *H*_*t*1_, and *T*) that induce inelastic deviations from hyperelasticity.

## Results

**Figure 8:**
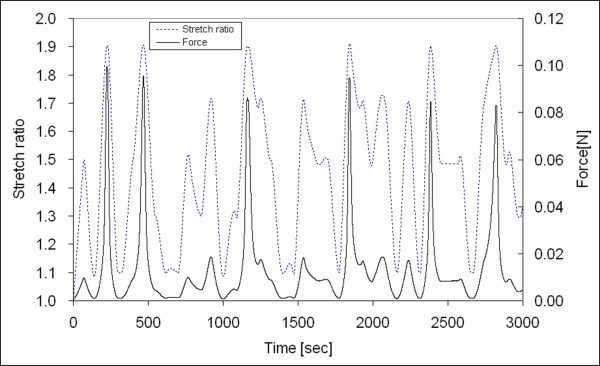
The randomized stretch-controlled protocol and corresponding force output profile for the tissue specimen (0–3000 sec).

**Figure 9:**
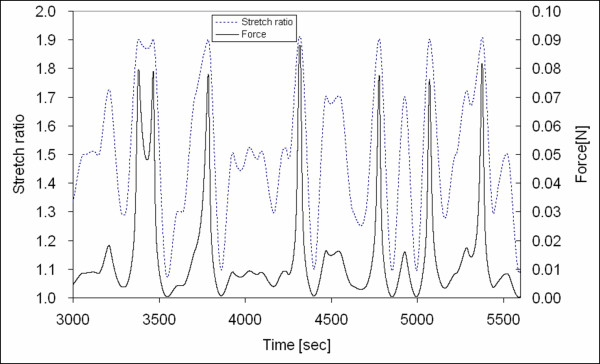
The randomized stretch-controlled protocol and corresponding force output profile for the tissue specimen (3000–5600 sec).

**Figure 10:**
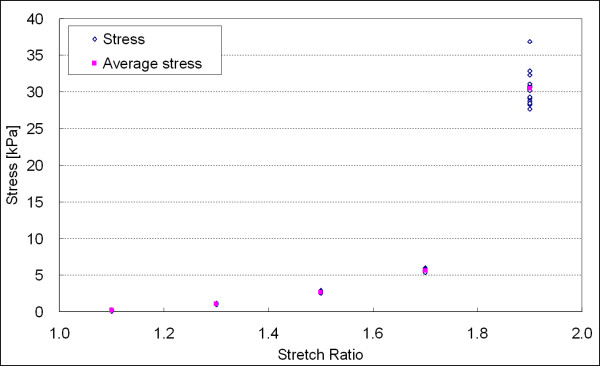
Stress versus stretch curve as obtained from measurements at the time nodes. The average at each nominal value of stretch is defined herein as the elastic part of the response. The deviation from the average is defined as the inelastic part or inelastic deviation.

The randomized stretch-controlled protocol and corresponding force output is plotted in Figures 8 and 9. Figure 10 shows the stress versus stretch. The square dots in Figure 10 represent the hyperelastic stress responses. The fractional uncertainty *FU *that represents the inelastic response for the stretch ratios *λ *= 1.1, 1.3, 1.5, 1.7, and 1.9 were 30.6%, 6.7%, 4.4%, 3.6%, and 8.1%, respectively. Although inelastic deviation from hyperelasticity increased to about 8.1% at maximum deformation (*λ *= 1.9), the inelastic deviation has a significant decreasing trend with increasing stretch ratio in the range of 1.1–1.7. Note that the amount of inelasticity was highest at the lowest deformation of interest (*λ *= 1.1). The fractional uncertainties corresponding to each stretch ratio are summarized in Table 1.

**Table 1:** Absolute and fractional uncertainties for each stretch ratio.

	1.1	1.3	1.5	1.7	1.9
*F*_*W *_[mN]	0.927	4.120	8.820	16.510	80.210
*D *[mN]	0.281	0.275	0.385	0.593	6.520
*FU *[%]	30.64	6.67	4.37	3.59	8.13

Based on the results of MLRA, *H*_*t*2_, *H*_*t*1_, and *T *were determinant factors for the inelastic deviation for most of the deformation ranges. They were, however, not equally effective for all deformation ranges. The parameter *T *was an effective factor for all deformations. Inelastic behavior at *λ *= 1.1 and 1.3 was solely determined by *T*. The effect of a stretch rate represented by short-term stretch history *H*_*t*2 _was more significant than other factors at *λ *= 1.5, 1.7, and 1.9. Moreover, its significance was greater at larger deformation. The long-term stretch history *H*_*t*1 _was moderately effective at *λ *= 1.5 and 1.9. The statistical models for *λ *= 1.1, 1.3, 1.5, and 1.9 showed high ${\bar{R}}^{2}$ values (above 85%). The ${\bar{R}}^{2}$ value of the statistical model for *λ *= 1.7 (${\bar{R}}^{2}$ = 71%) was lower than other cases but so was the fractional uncertainty itself. The statistical models obtained by MLRA for the various deformations of the tissue are summarized in table 2.

**Table 2:** Multivariable linear regression models for the tissue (*t*_*c *_is for two-tailed test).

*λ*	*Ind.var*	$\hat{\beta }$	*s*_*e*_	*t-*value	*t*_*c*(*α*/2;*n*-*k*-1)_, *α *= 0.1	${\bar{R}}^{2}$
1.1	$\hat{\alpha }$	0.34	0.03	10.44	1.75	0.92
	*T*	-1.70	0.13	-13.09		
	${\hat{D}}_{1.1}=0.34-1.70T$					
1.3	$\hat{\alpha }$	0.64	0.09	6.79	1.83	0.87
	*T*	-2.29	0.30	-7.72		
	${\hat{D}}_{1.3}=0.64-2.29T$					
1.5	$\hat{\alpha }$	5.85	1.65	3.55	1.77	0.85
	*T*	-1.00	0.20	-5.02		
	$H_{t_{2}}$	-17.48	8.15	-2.15		
	$H_{t_{1}}$	-5.75	2.63	-2.18		
	${\hat{D}}_{1.5}=5.85-T-17.48H_{t_{2}}-5.75H_{t_{1}}$					
1.7	$\hat{\alpha }$	10.42	2.96	3.52	1.81	0.71
	*T*	-0.97	0.35	-2.77		
	$H_{t_{2}}$	-36.77	10.76	-3.42		
	${\hat{D}}_{1.7}=10.42-0.97T-36.77H_{t_{2}}$					
1.9	$\hat{\alpha }$	13.56	1.91	7.10	1.83	0.98
	*T*	-1.58	0.09	-16.72		
	$H_{t_{2}}$	-44.76	6.46	-6.93		
	$H_{t_{1}}$	-2.77	0.94	-2.96		
	${\hat{D}}_{1.9}=13.56-1.58T-44.76H_{t_{2}}-2.77H_{t_{1}}$					

**Figure 11:**
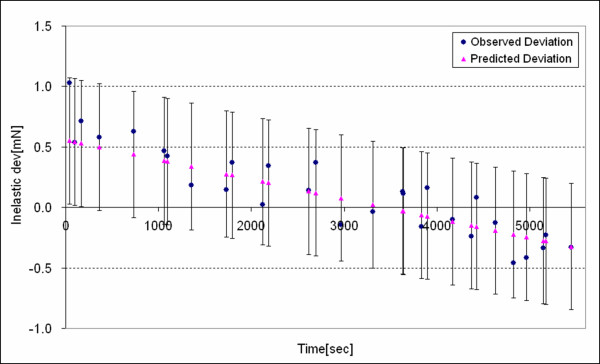
The observed inelastic deviation *D*_*i *_and predicted (or point forecasting) deviation ${\hat{D}}_{f}$ for *λ *= 1.3 of the tissue where *t*_*c*(*α*/2;*n*-*k*-1)_·*s*_*f *_= 0.523 [mN]. Lines represent standard deviations.

**Figure 12:**
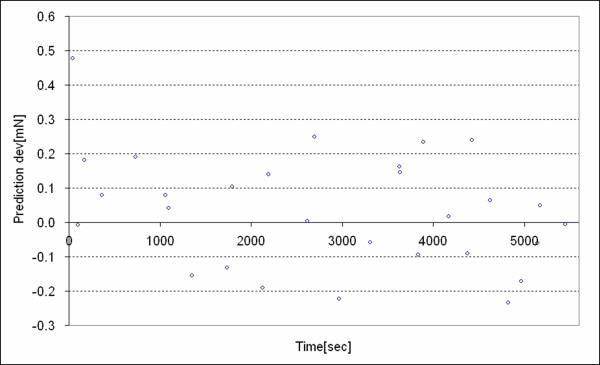
Distribution of the prediction deviation for *λ *= 1.3 of the tissue, versus *T*.

We found that the *deviation of the predication *was not systematic relative to our independent variables because the trends of the observed deviation were captured by the predicted deviation. For example, consider *λ *= 1.3 wherewith the observed inelastic deviation and the predicted inelastic deviation are plotted in Figure 11 and the deviation of the prediction is plotted in Figure 12. Inelastic deviation only depends on *T *for this stretch and both the observed deviation and the predicted deviation display this trend. The deviation of the prediction appears random with respect to *T*. Deviation of the predication for the other nominal stretch values with dependence on *H*_*t*2 _and *H*_*t*1 _were checked likewise and they also appeared as random or unsystematic with respect to our dependent variables.

## Discussion

This study characterizes the elastic (or mean) response and the inelastic (or deviation from the mean) response of a porcine pulmonary artery strip that underwent high strain with random stretch and stretch-rate prescribed. The inelastic behavior was most prominent for small deformations and primarily dependent on *T*, the time since the start of testing. The inelasticity at *λ *= 1.1 was almost 850% greater than that of *λ *= 1.7, and 400% greater than that of *λ *= 1.9. This finding can provide one reason for much ambiguity in the hyperelastic modeling for soft tissues at small deformations. The most important configuration for elasticity (i.e., the stress free reference configuration) is in the deformation region wherein inelasticity predominates. How the sample was prepared and any significant stretch history during dissection are likely to affect greatly the reference configuration and any subsequent constitutive relation that depends on the reference configuration. At the highest deformation *λ *= 1.9, inelastic deviation was increased again, and it is significant that in this specimen, this stretch is at the point where the stiffness starts to increase greatly. We suspect that this break in trend is due to complex microstructural coupling of fibers in radial and circumferential directions and some reorganizing of the fibers with the high deformation. Except for low deformation, the inelastic deviations for the other cases are less than 10%. Thus, an elastic model is mostly, say 90%, accurate for this strip of pulmonary artery.

MLRA clearly indicates that *H*_*t*2_, *H*_*t*1_, and *T *are determinant factors that induce inelastic behavior, but they are not equally influential throughout the stretch domain. Inelasticity of the tissue was dominated by different factors at different stretches, and it was systematic rather than random. *T *was an influential factor for all deformations, but particularly for low deformations. As the deformation increases, however, stretch rate is the most influential for the inelastic response. Given that our protocol randomized the stretch, it is not very significant that the deviation of the predication appears random or unsystematic after we correct for deviations due to *H*_*t*2_, *H*_*t*1_, and *T*.

In summary, we found that the stretch rate, stretch history, and time are important factors that give rise to the inelastic mechanical response of a strip of pulmonary artery as evident in a randomized uniaxial stretch-controlled stretch test. Their contributions were not the same for all stretches. This study was limited to one soft tissue specimen at room temperature; however, it is the opinion of the authors that one must be able to define the phenomenological behavior of one specimen before it is possible to define relations that represent the aggregate or typical behavior of a tissue. For example, if one could not measure the elastic modulus of a single sample of steel wire, one could not measure the average elastic modulus of steel. Albeit generalized, constitutive relations for particular tissues are mostly accurate and in use already; there is much ambiguity in their definitions. The focus of this work is determining the behavior of one sample, and in so doing, we hope to eventually define quantitatively how specimens are similar, and more importantly for exploiting natural variations in ultrastructure, how they differ.
